# Strategic partnerships to improve surgical care in the Asia–Pacific region: proceedings

**DOI:** 10.1186/s12919-023-00257-y

**Published:** 2023-07-25

**Authors:** Rennie X. Qin, Makela Stankey, Anusha Jayaram, Zachary G. Fowler, Sangchul Yoon, David Watters, Adrian W. Gelb, Kee B. Park

**Affiliations:** 1 The Program in Global Surgery and Social Change, the Department of Global Health and Social Medicine, Harvard Medical School, 641 Huntington Ave, Boston, MA 02115 USA; 2 Keck School of Medicine at the University of Southern California, 1975 Zonal Ave, Los Angeles, CA 90033 USA; 3 Tufts University School of Medicine, 145 Harrison Ave, Boston, MA 02111 USA; 4 Department of Medical Humanities and Social Sciences, College of Medicine, Yonsei University, Seoul, South Korea; 5 Faculty of Health, School of Medicine, Deakin University, Bellerine St, Geelong, VIC 3220 Australia; 6 Department of Anesthesia and Perioperative Care, University of California San Francisco, 521 Parnassus Ave, San Francisco, CA 94143 USA

**Keywords:** Global surgery, Surgical system strengthening, National surgical planning, Partnership, Asia–Pacific

## Abstract

Emergency and essential surgery is a critical component of universal health coverage. Session three of the three-part virtual meeting series on *Strategic Planning to Improve Surgical, Obstetric, Anaesthesia, and Trauma Care in the Asia–Pacific Region* focused on strategic partnerships. During this session, a range of partner organisations, including intergovernmental organisations, professional associations, academic and research institutions, non-governmental organisations, and the private sector provided an update on their work in surgical system strengthening in the Asia–Pacific region. Partner organisations could provide technical and implementation support for National Surgical, Obstetric, and Anaesthesia Planning (NSOAP) in a number of areas, including workforce strengthening, capacity building, guideline development, monitoring and evaluation, and service delivery. Participants emphasised the importance of several forms of strategic collaboration: 1) collaboration across the spectrum of care between emergency, critical, and surgical care, which share many common underlying health system requirements; 2) interprofessional collaboration between surgery, obstetrics, anaesthesia, diagnostics, nursing, midwifery among other professions; 3) regional collaboration, particularly between Pacific Island Countries, and 4) South-South collaboration between low- and middle-income countries (LMICs) in mutual knowledge sharing. Partnerships between high-income countries (HIC) and LMIC organisations must include LMIC participants at a governance level for shared decision-making. Areas for joint action that emerged in the discussion included coordinated advocacy efforts to generate political view, developing common monitoring and evaluation frameworks, and utilising remote technology for workforce development and service delivery.

## Introduction

In the third and final session of the three-part meeting series on *Strategic Planning to Improve Surgical, Obstetric, Anaesthesia, and Trauma Care in the Asia–Pacific Region*, 161 participants across 32 countries discussed the role of strategic partnerships. The session was opened by *Paul Farmer, Kolokotrones University Professor and Chair of the Department of Global Health and Social Medicine at Harvard Medical School.* He urged participants to not only be ‘stake holders’ but also ‘stake planters’, who stake their claim to improve surgical care. Framing statements on different forms of collaboration were followed by presentations by a range of partner organisations, including intergovernmental organisations, professional associations, academic institutions, non-governmental organisations, and the private sector. Table 1 summarises each partner organisation’s key messages and areas of support. Participants discussed various modes of collaboration, such as collaboration across the spectrum of care, interprofessional collaboration, regional collaboration, and South-South collaboration (Table 2). The session was moderated by *David Watters, Alfred Deakin Professor of Surgery at Deakin University, Australia, and Past President of Royal Australasian College of Surgeons (RACS)*; and *Adrian W. Gelb, Professor (emeritus) in the Department of Anaesthesia and Peri-operative Care, University of California San Francisco, US, and President of the World Federation of Societies of Anaesthesiologists (WFSA).*

**Table 1:** Partner organisations’ key messages and areas of work

Organisation type	Organisation name	Region/country	Key messages and areas of work by health system domain
Inter-governmental organisation	WHO Integrated Health Services Department	Global	• Ecosystem approach to surgical care congruent with the primary health care for UHC narrative
	UNITAR	Global	*Workforce:* high-level learning solutions, a platform for online courses, NSOAP workshop
	SPC	Pacific	• The importance of regional collaboration & leadership in advocacy, training, and NSOAP development • Sustaining support during NSOAP implementation
Professional associations	WFSA	Global	• Adopting the WHO-WFSA International Standards for a Safe Practice of Anaesthesia • Anaesthetists should be at the table when NSOAPs are developed
	RACS	Australia, New Zealand, the Pacific, Timor Leste, Myanmar	• Pivoting to remote education during the COVID-19 pandemic *Service delivery:* visiting specialists *Infrastructure:* equipment donation *Workforce:* training, scholarships, online education, training governance & regulation, curriculum development, competence-based training
	RANZCOG	Australia, New Zealand, the Pacific	• The importance of advancing surgical care in managing obstetric complications and cervical cancer • Innovative capacity-building solutions, such as distance and flexible learning • The need for collaboration across disciplines *Governance:* guideline creation *Workforce:* capacity building
	GICS	Global	• Unique surgical needs of children *Governance:* Optimum Resources for Children’s Surgery guideline, technical advice on integrating paediatric surgery into NSOAPs
Academic & research institutions	WHO CC Mongolia	Mongolia	*Service delivery:* development of minimally invasive and liver transplantation services
	WHO CC Mumbai	India	• The importance of South-South collaboration • The need to strengthen financing, leadership, and governance in NSOAPs *Information management:* trauma care, blood access, and workforce capacity research
	Fiji National University	Pacific	• Regional collaboration in training surgical, obstetric, and anaesthesia providers in the Pacific • The need to develop in-country training
	Lancet Diagnostics Commission	Global	• The importance of diagnostics to surgical and obstetric care • The role of pathologists in service delivery and guideline and policy development in global surgery
Non-governmental organisation	Interplast Australia & New Zealand	Australia, New Zealand, 18 countries in the Asia–Pacific	*Service delivery:* plastics and reconstructive surgery *Workforce:* capacity building, online support
	Health volunteer overseas	United States, Cambodia, Bhutan, Laos	*Workforce:* developing degree and residency programs, simulation training *Governance:* guideline development
Private sector	Johnson & Johnson	Asia–Pacific	*Infrastructure:* providing equipment and supplies *Workforce:* education, capacity building, access to digital education

**Table 2:** Modes of collaboration to advance surgical, obstetric, and anaesthesia care

Collaboration across the spectrum of care	Collaboration between surgery, obstetrics, anaesthesia, emergency medicine, intensive care and diagnostics, among other specialties to form an ‘ecosystem’ of care
Regional collaboration	Collaboration among countries in a defined geographic region to address shared challenges and generate joint solutions
Interprofessional collaboration	Collaboration among healthcare professionals of different backgrounds, such as medical specialists, nurses, midwives, trainees, and students
South-South collaboration	Collaboration among two or more low- and middle-income countries to exchange knowledge and resources

## Collaboration across the spectrum of care

### Ecosystem approach to advancing surgical care 

*Teri Reynolds, Head of the Clinical Services and Systems Unit, Integrated Health Services department, WHO,* discussed applying an ecosystem approach to global surgery. The primary health care (PHC) for universal health coverage (UHC) narrative is currently central in global health. PHC is broader than primary care itself; it encompasses a systemic approach to meet people’s needs at the primary care level. Primary, preventative, and promotive services are at the centre of this ecosystem; however, they must be surrounded by other services, such as emergency and surgical services. The term ‘ecosystem’ acknowledges the differences and yet complementary relationships between various services. Certain services, such as surgical care, must be delivered at and linked to hospitals to ensure safety. Whilst surgery performed in an operating theatre is not clinic-based primary care, it is encompassed by the ecosystem and linked to clinic-based primary care through mechanisms such as transportation, communication protocol, referral and counter referral systems. Therefore, it is necessary to both broaden the understanding of surgical care and maintain the specificity of service delivered in operating theatres, which have human resource, capacity, and material resource requirements.

The ecosystem concept presents surgical system strengthening to policymakers in a manner congruent with the PHC for UHC narrative. The COVID-19 pandemic has placed greater focus on critical care. The way each country creates boundaries between aspects of care, such as emergency, surgical, critical or anaesthesia care, varies worldwide. The ecosystem approach integrates the relationships among these forms of care, which are distinct from clinic-based promotive care. Linking global surgery to well-established health agendas, such as maternal and child health, injury, and road safety, is critical for its advancement.

## Regional collaboration 

*Berlin Kafoa,Team Leader, Clinical Services Program, Public Health Division, Pacific Community (SPC)* highlighted the importance of leadership and regional collaboration in Pacific Island Countries (PICs). Partnership with regional professional societies and academic institutions, such as Fiji National University and the University of Papua New Guinea, helped train the surgical, obstetric, and anaesthetic (SOA) workforce [1, 2]. Through regional collaboration and leadership, PICs advocated for surgical system strengthening at key forums, such as the Pacific Health Ministers Meeting, the WHO Regional Committee Meetings, and the World Health Assembly [3, 4]. Leadership and regional collaboration hold nations accountable for developing and implementing their National Surgical, Obstetric, and Anaesthesia Plans (NSOAPs). NSOAP implementation is far more complex than NSOAP development. Partners should be sure to maintain engagement with nations throughout implementation rather than withdrawing once NSOAP development is complete.

## Intergovernmental organisation 

### United Nations Institute for Training and Research (UNITAR)

*Geoff Ibbotson, Senior Health Consultant at UNITAR and Executive Lead of the Global Surgery Foundation* discussed UNITAR’s aim to build capacity within governments by bringing high-level learning solutions and services for country-level implementation. For example, UNITAR works to build global surgical capacity through hosting intensive NSOAP workshops for national leaders. In 2019, UNITAR offered to serve as the UN host for the Global Surgery Foundation. UNITAR and the Global Surgery Foundation can serve as common platforms for online courses to extend program reach and maximise uptake. Another example is the Trauma Resuscitation in Kids program, created by the Royal College of Physicians and Surgeons of Canada, that will soon be added to the UNITAR platform.

## Professional associations 

### Royal Australasian College of Surgeons (RACS)

*Annette Holian, orthopaedic and trauma surgeon at Monash Children’s Hospital, Australia, and Councillor at RACS* stated that RACS is a single surgical college across two countries, Australia and New Zealand and encompassing nine surgical specialties. Technical expertise is only one of the ten surgical competencies taught at RACS. Cultural competency and cultural safety is the most recently added competencies, particularly important for surgeons working overseas. RACS Global Health is primarily funded through the Australian Department of Foreign Affairs and Trade (DFAT) and donations. All international work done by RACS is delivered pro-bono by RACS fellows and other professional collaborators. This work includes service delivery support, training, equipment donation, monitoring and evaluation, and scholarships [2, 5, 6]. Moving forward, RACS is transitioning from specialist service and provision of education towards more capacity building. Thus, they have established a new online educational initiative in collaboration with other colleges in the Pacific region. The COVID-19 pandemic has required developing enhanced audio-visual facilities with live, synchronous, remote education, to support learners in their own environments. RACS could support additional areas, including training governance and regulation, curriculum development, competency-based training, exit exams, and transition to practice. RACS’ priorities are driven by its partners’ needs. Online surgical training and career-long learning in the ten RACS competencies are key areas where RACS can contribute.

### World Federation of Societies of Anaesthesiologists (WFSA)

*Wayne Morriss, President-Elect, WFSA* discussed WFSA's role as a global network representing several hundred thousand anaesthetists in 150 countries. WFSA works with national societies and other organisations in advocacy, education and training, and patient safety. WFSA has an official liaison role within WHO and works closely with surgical colleagues to represent safe anaesthesia and surgery on the global health agenda. UHC will only be achieved through access to safe surgery; safe surgery relies on safe anaesthesia. If surgery is the neglected stepchild of global public health, then anaesthesia is surgery’s invisible friend. The public awareness of anaesthesia is often low; anaesthesia advocates rely on the surgical community as allies. However, anaesthesia should be recognised as a medical specialty rather than an appendage of surgery. Unfortunately, some countries have developed surgical subspecialties without providing accompanying resources for anaesthesia care. Anaesthesia plays a vital role in areas other than surgery, such as pain management and critical care. Inadequate resources for anaesthesia should not become the rate-limiting factor in surgical system strengthening, nor should the landscape of anaesthesia and surgical care be thought of as a zero-sum game. Both strong anaesthesia and surgical care are needed. WFSA continuously works with national societies to advocate for universal access to safe anaesthesia; however, assistance is needed in two areas. First, the WHO-WFSA *International Standards for a Safe Practice of Anaesthesia* should be adopted in all countries that don’t already have adequate standards for safe anaesthesia care [7]. These guidelines are endorsed by WHO and offer a graded approach to strengthening anaesthesia in low-resource environments. Second, anaesthetists should be at the table when NSOAPs are developed. Without input from anaesthetists, NSOAPs may not achieve their desired outcomes.

### Royal Australian and New Zealand College of Obstetricians and Gynaecologists (RANZCOG)

RANZCOG is a non-profit professional organisation dedicated to establishing the highest standards of practice in women’s health and providing clinical and technical support for obstetricians and gynaecologists in the Asia–Pacific region. *Leeane Panisi, head of obstetrics and gynaecology, National Referral Hospital, Solomon Islands* and *Rebecca Mitchell, obstetrician and gynaecologist at Sunshine Hospital, Australia, and member of the RANZCOG Global Health Committee,* provided an example of successful partnership in guideline creation. Mitchell and Panisi collaborated to develop guidelines for healthcare workers across the Solomon Islands to reduce preventable maternal mortality and morbidity. They formed a working group comprising obstetricians, gynaecologists, midwives, and registrars, and developed a pocket-sized book distributed to all healthcare providers in the Solomon Islands. The book has helped standardise and spread specialist knowledge throughout the Solomon Islands.

*Amanda Noovao-Hill, Head of Department for Surgery, Anaesthesia, Obstetrics and Gynaecology, and Ophthalmology at Fiji National University, co-founder of the Pacific Island Cervical Cancer Screening Initiative*, *and member of the RANZCOG Global Health Committee* spoke about the importance of surgical care in obstetrics and gynaecology. She explained that obstetricians primarily focus on maternity care, of which surgical care constitutes a small part. However, saving mothers’ lives occasionally requires advanced surgical skills, which many obstetricians may not possess, such as a difficult Caesarean section with a low-lying anterior placenta. In the Pacific, there is a high prevalence of cervical cancer, yet radiotherapy is unavailable [8]. Surgical options are optimal for women presenting with early disease, but they require specialised gynaecologic oncology skills unavailable in the Pacific. Recently, Fiji held its NSOAP stakeholder meeting. Governance, workforce capacity, financing, and training were discussed at length. In low-resource settings, the sharing of skills, equipment, and training opportunities must be optimised; however, there is often a preoccupation with discipline-specific challenges. The RACS Pacific Island Program (PIP), Strengthening Specialist Clinical Services in the Pacific (SSCSiP), and RANZCOG are key supporters of surgical care in Fiji and the Pacific region. However, the support of these organisations has not brought about sufficient capacity building. The increasing retirement of senior and experienced surgical staff has exacerbated staffing shortages. Noovao-Hill shared a number of sustainable, practical strategies for capacity building in the Pacific region, including collaborative training, resource sharing, distant and flexible learning supported by targeted visits, and the development of local good-quality skill labs. She called for the support of HIC colleagues to work in equal partnership with local leaders.

### Global Initiative for Children’s Surgery (GICS)

*Tahmina Banu, Director of Chittagong Research Institute for Children Surgery and board member of GICS*discussed the role of GICS is a global consortium of providers, institutions, and allies from both low- and middle-income countries (LMICs) and high-income countries (HICs) involved in children’s surgical care. The consortium includes paediatric surgeons, anaesthetists, nurses, radiologists, pathologists, and advocates. Children make up as much as 50% of the population in many countries, particularly LMICs of the Asia–Pacific region; however, two-thirds of the world’s children lack access to surgical care. All age groups of children: neonates, infants, and adolescents, have their own unique surgical needs that differ from adults. Children’s surgery is generally not included in NSOAPs. GICS has developed *Optimal Resources for Children’s Surgery* = guidelines for children’s surgery, stratified by subspecialty, level of care, and patient age [9]. The guidelines also document the required personnel, equipment, facilities, procedures, training, research and quality improvement components at each healthcare level [10]. They could serve as a benchmark to integrate paediatric surgical care capacity building into NSOAPs.

## Academic and research institutions

### Fiji National University (FNU)

*William May, Dean, College of Medicine, Nursing, and Health Sciences, FNU *stated that FNU was established in 1885. Its postgraduate clinical programs and public health programs were established in 1998. In the past five years, the school has trained 128 postgraduate students. 31% of the school’s graduates are trained in surgery. Despite this long history of workforce training, ongoing improvements are required. The school faces challenges in recruiting, retaining, and sustaining its current workforce. Regional students from Pacific Island Countries leave behind workforce gaps in their home countries when they travel to Fiji for training. Where supervisors and caseloads allow, FNU facilitates in-country attachments. It has done so for some Pacific Island Countries and hopes to expand this in the future.

### WHO CC for reference on emergency and essential surgical care, Mongolia 

*Sergelen Orgoi, senior surgeon at the First Central Hospital of Mongolia and Head of the WHO CC, explained that* Mongolia has worked with various international collaborators, including Swiss Surgical Teams, Swanson Family Foundation, Center for Global Health at the University of Utah, Society of American Gastrointestinal and Endoscopic Surgeons, Asan Medical Center in South Korea, American College of Surgeons, and the Australian embassy. Through this collaboration, Mongolia has developed access to laparoscopic cholecystectomy and liver transplant services with excellent clinical outcomes [11].

### WHO CC for research in surgical care delivery in LMICs, India 

*Nobhojit* *Roy, Director of Health System Strengthening at CARE-India,* described three thematic areas within the WHO CC: 1) volume and burden of surgically treatable diseases, 2) trauma care quality and outcomes, and 3) cancer care [12]. The WHO CC aims to strengthen surgical systems in partnership with the Indian university hospitals and research consortium (IND-Surg). In urban areas, trauma care is a focus topic supported by bilateral grants. National trauma data management systems and registries have been established [13]. In rural areas, projects include mapping blood access in Bihar and examining workforce capacity as a determinant of Caesarean section rate [14, 15]. The WHO CC has strong South-to-South collaboration within the South Asia region. They have worked with Nepal and Bangladesh to address breast and cervical cancer care. Among the six WHO building blocks of NSOAPs: good progress is being made in India in service delivery, infrastructure, workforce, and health information; however, health financing and leadership, management, and governance must still be addressed.

## Non-governmental organisations

### Interplast Australia and New Zealand

*Michael McGlynn, President of Interplast Australia and New Zealand* stated that Interplast is a non-profit organisation established in 1983 by RACS in partnership with Rotary. The organisation operates on donations and volunteers, sending teams of surgeons, anaesthetists, nurses, and allied health professionals by request to 18 countries in the Asia–Pacific region over 40 times each year. They work in partnership with local organisations to alleviate physical disability and deformities caused by congenital abnormalities, trauma, burns, cancer, and disease through appropriate surgical interventions. Before the COVID-19 pandemic, two-thirds of their work focused on training and mentoring and a third on service delivery. During the COVID-19 pandemic, Interplast has been unable to provide on-site support, and an online support system was piloted. Since 2020, they have conducted over 40 webinar sessions for over 2,500 clinicians and provided numerous single-site meetings, case conferences, and one-on-one mentoring sessions.

## Interprofessional collaboration

### The role of diagnostics

*Annie Cheung, Laurence L.T. Hou Professor in Anatomical Molecular Pathology at the University of Hong Kong and member of the Lancet Commission on Diagnostics*, discussed the pivotal role of pathology and laboratory medicine in surgical and obstetric care [16]. The geographic variation in incidence, mortality and morbidity of various diseases is linked to the capacity to prevent, diagnose, treat, and monitor these diseases. For instance, cross-country variation in 5-year net survival for childhood cancers correlates to the in-country diagnostic capacity [17]. Although cervical cancer is preventable, its prevention, diagnosis, and treatment remain challenging in many countries. Lack of access to pathology and laboratory services is a substantial barrier in LMIC [18]. Pathology and laboratory science have made significant advances in the past century, particularly in molecular cancer diagnostics [19]. However, access to diagnostics varies by geography and population, even within a single country. In some settings, genetic pathology is routinely applied in disease diagnosis and management [20]; however, other locations may even lack reliable cross-matching and blood bank services [21]. Health management leaders and donors should recognise the essential role of pathology and laboratory medicine. Pathologists must be involved in all levels of planning. Their involvement could help government steering committees to address specific diseases, such as cancer prevention, professional associations to establish good practice guidelines, and charities to support medical tests in LMICs.

### The role of nurses in task shifting

*Richard Henker, Professor, Department of Nurse Anaesthesia, University of Pittsburgh,* discussed his work with Health Volunteers Overseas (HVO) to support nurse anaesthesia programs in Cambodia, Bhutan, and Laos [22]. HVO volunteers have worked collaboratively with nurse anaesthetists from Phnom Pen and Bangkok and the Ministry of Health to develop various programs in these three countries. These programs include nursing anaesthesia bachelor degree programs, scholarship for overseas certificate programs in Bangkok, a nurse anaesthetist residency program, crisis team performance simulation training, and standards of practice for nursing and infection prevention and control. Their work is also supported by the WFSA Bangkok Anaesthesia Regional Training Center (BARTC) program.

Nurse anaesthetists play a vital role in Laos, where 652 surgeries are performed per 100,000 population annually. Laos has 495 physicians and 60 anaesthetists. Nurses, medical assistants, and general physicians provide 70% of anaesthesia care in Laos. Future plans for improving anaesthesia in Laos include conducting an anaesthesia workforce assessment, supporting the residency program at Mahosod Hospital, and reopening the nurse anaesthetist program at the University of Health Sciences.

## The private sector

### Johnson and Johnson

*Ashish Kohli, Senior Director, EndoMechanical Platform, Johnson and Johnson Medical Devices Companies* discussed Johnson and Johnson’s global health work in Asia–Pacific region. This includes supply provision, education, and capacity building. They work with community organisations to reduce maternal and infant mortality. They have collaborated with Operation Smile to provide care to children with cleft lip and palate for the past 30 years. Johnson and Johnson has recently extended digital access to education for surgeons and health professionals.

## Discussion

### The importance of obstetrics in surgical system strengthening 

Participants acknowledged the importance of obstetrics in surgical system strengthening and discussed key performance indicators in obstetric care. Whilst maternal mortality rate is measured in many settings, it is not included as a Lancet Commission on Global Surgery (LoGS) indicator.

*Ravichandran Jeganathan, National Head of Obstetrics and Gynaecological Services at the Ministry of Health, Malaysia*, stressed the importance of having robust obstetric data to inform intervention design. Malaysia started a national obstetric registry in 2009, with 1.3 million datasets collected to date [23]. During the COVID-19 pandemic, they have discovered two ‘bright spots’. Firstly, even though patients could not be moved to referral hospitals due to COVID restrictions, specialists could travel to district hospitals to provide training and strengthen district hospital capacity. Secondly, protocols became modified to be more patient-centred. Through providing adequate care and training, Malaysia has decreased the number of unnecessary surgeries to treat the complications of inadequate maternal care, such as third-degree tear repair.

Mitchell echoed the importance of obstetric data collection, which is a challenge for many LMICs. Maternal mortality rates are complex indicators affected by many factors, including access to contraception and family-planning tools. The greater the need for family planning, the greater the maternal mortality rate. Access to surgery is similarly complex, relying on many factors, including access to blood, pathology services, and sterilisation.

Participants highlighted the importance of standardised templates for collecting obstetric data. Noovao-Hill said that the methods for collecting data are slightly different at each centre in Fiji. They realised that data needs to be collected in a uniform way to be comparable on a national level. Ravichandran said that WHO does have a standardised template for maternal death reporting [24]. He is very willing to share Malaysia’s data and experience in maintaining a database as an LMIC. Political will, local champions, and an anonymous common platform could facilitate data sharing between countries.

Participants commented that midwives and obstetricians work together to improve maternal outcomes. However, this collaboration may vary in some settings. Improving maternal mortality often relies upon improving relationships among medical professions and specialties. Similarly, poor outcomes may result from the lack of collaboration between specialties.

### Creating the political will 

Participants discussed the challenge of creating the political will for surgical system strengthening. Lord Viliami Tangi shared insight from his experience as the chief surgeon and former Deputy Prime Minister of Tonga. The performance of leaders depends on their level of knowledge. Healthcare providers should take the time to educate their leaders. He met with his Minister of Health and gave a presentation on the importance of NSOAPs [25]. Kafoa added providing NSOAP advocacy and education at the regional level is necessary.

### Leveraging remote technology for surgical system strengthening

Participants discussed the use of virtual technology in service delivery and training.

Margaret Tarere-Lehi, *senior obstetric registrar, Vila Central Hospital, Vanuatu* advocated for the use of telehealth. This approach connects remote health workers to specialists in referral hospitals, increases access to specialist consultation, and promotes timely diagnosis and referral. Telehealth can build reliance in communities and connect health workers with their communities. Holian concurred and explained that Australia uses telehealth extensively for regional and rural communities. This approach is effective and cost- and time-saving. Interplast has conducted multiple virtual one-on-one case meetings and case conferences to plan procedures.

Participants discussed the benefits of virtual training initiatives. Increased online learning has created incredible opportunities for split faculty across sites. Recently, local faculty delivered paediatric life support training in Port Moresby, Papua New Guinea, with Australian faculty as remote backup. This partnership helped build capacity. Recorded training could afford non-native English speakers the opportunity to review English language content as needed. McGlynn shared that.

Ram Nataraja, *paediatric surgeon at Monash Children’s Hospital*, Australia, underscored the importance of simulation training. During the COVID-19 pandemic, he helped run a national surgical training program in Australia that put laparoscopic trainers in people’s homes. With motion tracking, doctors can remotely teach laparoscopic skills.

Speaking about the experience of WFSA, Morriss pointed out that additional funding and resources are required to effectively convert existing education programs into remote education programs. Remote learning tools must be specifically designed. Slightly modified digital versions of in-person teaching methods are inadequate for providing training and education virtually.

Kohli explained that Johnson and Johnson is experimenting with online proctorship. It is possible to remotely coordinate movements between a proctor and a trainee surgeon. Companies such as Johnson and Johnson could support these practices by developing, generating, or translating teaching materials.

### Cost of equipment and consumables

Participants pointed out that access to affordable surgical equipment and consumables is challenging for many LMICs and asked how medical device companies could address this issue. Kohli said that Johnson and Johnson is developing new cost-saving technologies, such as inhalable powdered oxytocin that does not require refrigeration.

### An international module-based curriculum 

Frank Piscioneri, *Clinical Director of Surgery, the Canberra Hospital, Australia,* proposed creating a global education needs analysis to track countries’ progress and determine their future goals. An international module-based tool could allow countries to select modules most critical for their needs. This program could provide resources to aid implementation beyond curriculum content. Linking many global training systems would increase training efficiency.

### Strengthening pathology capacity through remote technology and intersectoral collaboration

Watters emphasised the need for remote pathology assistance. It can take between six weeks and three months to receive a histology result in remote settings. Cheung reported that Cambodia only has six pathologists and inadequate facilities for processing and reporting. She suggested channelling pathology samples to nearby pathology labs. Alternately, she suggested disseminating the basic equipment for tissue processing so that images of tissue slides could be easily shared with remote partners. Kafoa pointed out that solutions to these kinds of challenges are often found in an unexpected manner. He urged that health needs, including pathology, be raised during trade agreement negotiations.

### Representation and shared decision making 

Kiki Maoate, *President of the Pasifika Medical Association (PMA), New Zealand,* suggested that colleges enhance representation by including board members from the Pacific at their governance level. This could reduce disparities, miscommunication, and duplications. Holian shared that RACS has restructured their global health program, engaging with clinical leads from throughout the region. This restructuring aims to create a global health program steering group where local clinical leads will participate in decision-making and represent their countries and institutions at the regional level. Maoate concurred with creating a steering group and reported that 22 countries in the region are already engaged in collaborative relationships that could be used to nominate delegates.

The PMA has had a presence in the Pacific region for over 20 years, supporting colleagues and countries with surge response to natural disasters, systems, and leadership. Ted Hughes, *anaesthetist, North Shore Hospital, New Zealand*, agreed that the PMA has been pivotal in advocating and gathering support for South Pacific nations. They have deployed staff from New Zealand during disasters and in response to other medical and surgical needs.

Debbie Sorensen, *Chief Executive Officer, PMA*, said that all colleges should increase the number of fellows in the Pacific, and fellows in the Pacific should be encouraged to engage in global health activities. RACS has a large number of fellows in the Pacific who could be mentored and supported to provide leadership and bridge the divide between nations and organisations. All partners in the region could apply this approach.

### Supporting the implementation of national surgical plans

A participant pointed out that Zambia was the first country to develop an NSOAP; however, there has been little international support for Zambia’s surgical plans. Developing an NSOAP is relatively easy; however, without financing and implementation, these plans can stagnate and fail to bring about changes.

Participants noted that there has been little support for NSOAP implementation once they are developed. Partners’ enthusiasm must be leveraged to maintain support throughout implementation. NSOAPs must be supported by targeted efforts to address gaps identified at the local level.

## Conclusion 

During this session, a diverse range of partner organisations discussed the role of strategic.partnership in advancing surgical, obstetric, and anaesthesia care in the Asia–Pacific region. Participants highlighted the importance of interprofessional partnerships across the spectrum of care to break specialty siloes. Obstetrics and anaesthesia are core pillars of the global surgery movement alongside surgery. Surgical system strengthening efforts in the Asia–Pacific region must be inclusive of voices from obstetrics and anaesthesia. Beyond the three core disciplines of global surgery, there is a need to build a broader ecosystem around surgical care by forging collaboration with a range of specialties across preventive, promotive, and curative care, such as emergency care and diagnostics. Not only do specialists play an important role but also nurses and midwives. Beyond the traditional model of North–South partnerships, participants highlighted regional partnerships and South-South partnerships between LMICs as critical modes of collaboration going forward. Participants discussed the opportunities for novel partnerships through remote training, mentorship, and service delivery during the COVID-19 pandemic.

The strength of this session Is the number and diversity of organisations represented. Not only is the quantity of partnerships important, but also their quality in terms of equity and sustainability. Participants discussed the issue of representation, governance, and power management in determining equity. However, given the time limit of this session, other factors affecting the equity and quality of partnerships are not fully explored. Future forums should explore the extent to which partners’ priorities align with the needs of patients and countries. Whilst most of the support provided by partners has traditionally been in the domain of workforce development, going forward, there is a need to be imaginative and look beyond the health sector to form intersectoral collaboration to address other important issues such as trade negotiations and surgical equipment access.

## Data Availability

N/A.

## Abbreviations

DFAT: Department of Foreign Affairs and Trade
GICS: Global Initiative for Children’s Surgery
HICs: High-income countries
LCoGS: Lancet Commission on Global Surgery
LMICs: Low- and middle-income countries
NSOAP: National Surgical, Obstetric, and Anaesthesia Plan
PMA: Pasifika Medical Association
PHC: Primary health care
PIC: Pacific Island Country
RACS: Royal Australasian College of Surgeons
RANZCOG: Royal Australian and New Zealand College of Obstetricians and Gynaecologists
SPC: Pacific Community
UHC: Universal health coverage
UNITAR: United Nations Institute for Training and Research
WFSA: World Federation of Societies of Anaesthesiologists
WHO: World Health Organization
WHOCC: World Health Organization Collaborating Center
